# Inflammatory Bowel Disease: Focus on Enteropathic Arthritis and Therapy

**DOI:** 10.2478/rir-2022-0012

**Published:** 2022-07-06

**Authors:** Amir Barkhodari, Kate E. Lee, Min Shen, Bo Shen, Qingping Yao

**Affiliations:** 1 Division of Rheumatology, Allergy and Immunology, Stony Brook University Renaissance School of Medicine, Stony Brook, NY, USA; 2 Center for Inflammatory Bowel Diseases, New York-Presbyterian/Columbia University Irving Medical Center, New York, NY, USA; 3 Department of Rheumatology and Clinical Immunology, Chinese Academy of Medical Sciences & Peking Union Medical College Hospital, Beijing, China

**Keywords:** autoimmune disease, autoinflammatory disorders, biologic, extraintestinal manifestations, inflammatory bowel disease, spondyloarthritis, Yao syndrome

## Abstract

Inflammatory bowel disease (IBD) is a chronic inflammatory disease primarily affecting the gastrointestinal (GI) tract and other organs. In this article, we provide a comprehensive review of IBD, particularly in the context of enteropathic arthritis and its therapeutic advances. Patients with IBD present with intestinal and extraintestinal manifestations (EIMs). Enteropathic arthritis or arthritis associated with IBD (Crohn's disease [CD] and ulcerative colitis [UC]) is the most common EIM and can involve both peripheral and axial joints with some overlaps. Furthermore, peripheral arthritis can be divided into two subcategories. Due to its varied inflammatory presentations and association with NOD2 mutations, CD can mimic other autoimmune and autoinflammatory diseases. Differential diagnosis should be extended to include another NOD2-associated disease, Yao syndrome. Therapy for IBD entails a myriad of medications and procedures, including various biologics targeting different pathways and Janus kinase (JAK) inhibitors. A better understanding of the therapeutic efficacy and mechanism of each drug aids in proper selection of more effective treatment for IBD and its associated inflammatory arthritis.

## Introduction

Inflammatory bowel diseases (IBDs), including Crohn's disease (CD) and ulcerative colitis (UC), are chronic inflammatory diseases of the gastrointestinal (GI) tract, with chronic or relapsing and remitting clinical courses.^[1]^ The etiology of IBD is not fully understood yet. Classic IBD is considered to be idiopathic with the contribution of genetic, environmental, microbiological, immunological, and metabolic factors, although the concept of secondary IBD with identifiable triggering factors has been reported.^[2]^ The conventional theory of the pathogenesis of IBD involves a dysbiotic shift, followed by dysregulated innate and adaptive immunity promoting inflammation in a genetically susceptible host.^[3]^ Patients with IBD have been found to have noticeable shifts in representations of particular bacterial taxa^[4]^ and genetic predisposition, such as mutations in the NOD2 gene.^[5]^ The pathogenesis for the development of arthritis associated with IBD remains elusive. Earlier literature examined the relationship between inflammatory arthritis and IBD. Progress has been slow in this regard. According to the current literature, microbiome dysregulation and its driven T helper 17 cell expansion and immune cell migration to the joint in a proper genetic background may play a role.^[6]^

IBD has varied presentations, and its diagnosis is made based on clinical, endoscopic, radiographic, and histologic findings of inflammatory and structural changes.^[1]^ There are certain distinguishing characteristics between CD and UC. In terms of GI manifestations, diarrhea is typically nonbloody in CD, while it is commonly bloody in UC. CD may involve any part of the GI tract and may have a segmental distribution with “skip lesions,” whereas UC is confined to the colon, rectum, or left-sided large bowel.^[1]^ Histologically, CD gut inflammation can extend transmurally, leading to strictures, fistulas, and abscesses as complications, while UC inflammation is typically confined to mucosal to superficial submucosal layers.^[1]^ Approximately 9% of patients initially diagnosed with UC or CD required a change in their diagnosis to each other within the first 2 years post-diagnosis.^[7]^ There is difficulty distinguishing CD and UC sometimes, and 10%–15% of patients carry a diagnosis of unclassified IBD.^[8,9,10]^ Terms such as IBD-indeterminate or indeterminate colitis are also used in clinical scenarios in which disease diagnosis remains unclear, despite a thorough GI workup. IBD patients are at higher risk for various autoimmune diseases, including rheumatoid arthritis, psoriasis, and celiac disease, than the general population.^[11]^ Occasionally, autoinflammatory diseases like familial Mediterranean fever can coexist with CD.^[12]^

In this review, we will focus on articular manifestation associated with IBD and its management.

## Extraintestinal Presentations of IBD With Focus on Arthropathy

### Extraintestinal Manifestations (EIMs) of IBD

EIMs of IBD can present in various ways. Further complicating the GI tract involvement is its slate of EIM involving the skin, eyes, joints, liver, lungs, and/or pancreas.^[13]^ EIMs include erythema nodosum, pyoderma gangrenosum, aphthous stomatitis, pyostomatitis vegetans, sweet syndrome, uveitis, episcleritis/scleritis, bronchiectasis, nephrolithiasis, glomerulonephritis, tubulointerstitial nephritis, amyloidosis, thromboembolic cerebrovascular disease, and primary sclerosing cholangitis.^[14,15,16]^

### Arthropathy Associated with IBD

Spondyloarthritis (SpA) is a group of inflammatory arthritides with shared clinical features, including axial spondylitis, sacroiliitis, peripheral inflammatory arthritis, enthesitis, and dactylitis. Enteropathic arthritis belongs to this group of diseases.^[17]^ Inflammatory arthritis is the most common EIM of IBD, with a prevalence 6%–46%.^[18]^ It can be peripheral, axial, or both, as commonly seen in other types of SpA.^[16]^ Peripheral joint involvement occurs in 5%–14% of patients with UC, and 10%–20% of those with CD. Axial involvement is seen more often in patients with CD than in those with UC and is more often associated with HLA-B27.^[19]^

Peripheral joint involvement is the most common articular presentation in IBD. Clinical characteristics are classified as two subtypes as follows.^[20]^

### Type 1

This type mostly presents as acute and pauciarticular arthritis involving the lower extremities (knee and ankle). It makes up to 5% of IBD patients, and 30% of them develop arthritis even before or in the early state of IBD. It is strongly associated with IBD activity and other systemic manifestations like erythema nodosum and uveitis. Synovial fluid is inflammatory with 5000–12,000 or up to 50,000 white blood cells/mm^3^ and predominantly neutrophils. The joint disease is self-limiting in 90% of patients and often resolves within 3–6 months. Radiographic changes or deformities are not common. Genetically, type I arthritis is associated with HLA-B27, B35, and DRβ1*0103.^[20]^

### Type 2

This type of presentation is more chronic mostly with symmetric polyarticular arthritis involving the metacarpophalangeal (MCP), knee, ankle, and other joints. The frequency of this type of arthritis is approximately 4% of IBD patients. It is known to be independent of IBD activity and does not correlate with extra-articular manifestations but can correlate with uveitis. Joint erosions and deformities can be seen. This type of arthritis is associated with HLA-B44.^[20]^ The axial joint can also be involved in IBD in a similar way to SpA, ranging from asymptomatic sacroiliitis to inflammatory low back pain with or without sacroiliitis to bilateral and symmetric sacroiliitis. Usually, symptoms occur insidiously with inflammatory low back pain.^[16]^ Sacroiliitis is usually bilateral with inflammatory low back and/or buttock pain. This type of joint disease has less association with HLA-B27 generally than axial SpA. Sacroiliitis also can be seen with or without spondylitis and can be asymptomatic.^[15]^ Other musculoskeletal manifestations associated with IBD are SAPHO syndrome,^[21]^ hypertrophic osteoarthropathy,^[22]^ dactylitis, and enthesitis.^[16,23]^

Articular manifestation can be different depending on the site of intestinal involvement. In CD patients with colonic involvement, arthritis is more frequent than isolated small bowel disease.^[24]^ Gut dysbiosis is more frequent in ankylosing spondylitis patients than in control patients, which is associated with worse axial SpA disease activity and physical function irrespective of both gut inflammation and treatments. This provides further evidence for an important link between disturbances in GI homeostasis and articular symptoms.^[25]^

## Differentiation Between Enteropathic Arthritis and Arthropathy Associated with Other Autoimmune/Autoinflammatory Diseases

Entropathic arthritis belonging to SpA shares many manifestations with ankylosing spondylitis/axial SpA and PsA. In rare instances, patients may present with a combination of inflammatory arthritis, colitis/ileitis, and psoriasis.^[26]^ In the absence of IBD manifestations, peripheral arthritis (specifically sub-type II, polyarticular) can be misdiagnosed as RA. However, peripheral arthritis in IBD is quite distinct from other inflammatory arthritis since there is little or no joint destruction, and tests for rheumatoid factor and antinuclear antibodies are generally negative.^[24]^ Behçet's syndrome (BS) is another great mimicker of IBD. BS is characterized by recurring and remitting of symptoms including oral aphthous ulcers, genital ulcers, uveitis, and cutaneous and GI manifestation. Any part of the GI tract from the esophagus to the rectum may be involved, but the most common location of intestinal Behçet's disease is the ileocecal area.^[27]^ BS and IBD share many clinical and endoscopic features, which makes it very challenging sometimes to differentiate the GI involvement of Behçet's disease from IBD.^[28]^

In autoinflammatory diseases, rash, arthralgia, and GI symptoms are common clinical features. Occasionally, patients with IBD may present with these symptoms as well as febrile episodes. Genetically, these patients may harbor genetic mutations in the MEFV gene as well. In this clinical scenario, patients may be diagnosed with both IBD and familial Mediterranean fever. Some patients may fulfill the diagnosis of CD but have atypical manifestation for FMF, or vice versa.^[29,30]^ Management of these combined conditions may involve therapy with colchicine and biologics.

**Figure 1:**
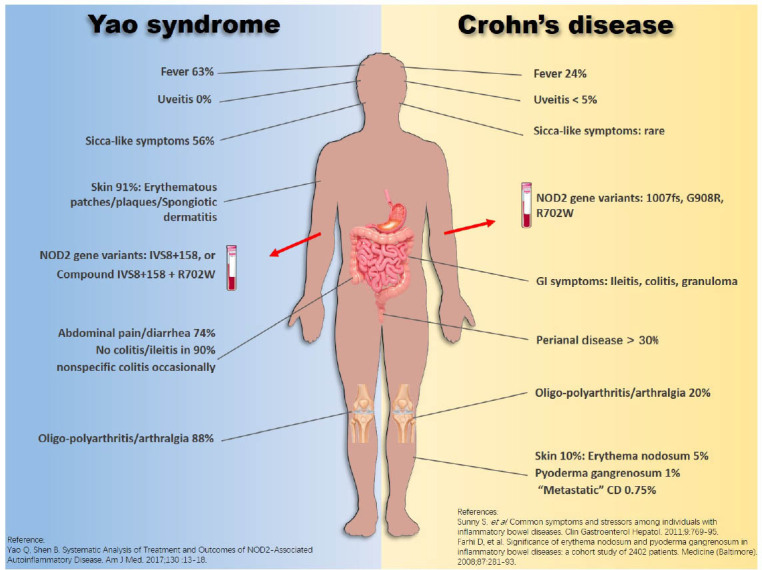
Phenotypic similarities and differences between Yao syndrome and Crohn's disease. CD, Crohn's disease.

Yao syndrome (YAOS, OMIM 617321) is a recently reported inflammatory disease. Its clinical manifestation can mimic CD, and both YAOS and CD share genetic mutations in the NOD2 gene. Herein, we briefly illustrate the similarities and differences between these two diseases (Figure 1).^[31]^ YAOS was formerly designated as NOD2-associated autoinflammatory disease.^[32]^ Since our initial report in 2013,^[33]^ the disease has been increasingly recognized in the field of medicine. YAOS does not appear uncommon and is predominantly reported in Caucasian adults, with a female-to-male ratio of 2:1. Classic cases present with recurrent fever, dermatitis, arthritis, distal extremity swelling, GI symptoms, and sicca-like symptoms/eyelid swelling. All YAOS patients carry NOD2 variants, with NOD2 IVS8+158 in 95%, and compound IVS8+158 and R702W in 30% or other NOD2 variants.^[34]^ Approximately 70% of YAOS patients have intermittent abdominal pain, bloating, and diarrhea. As a result, they often seek gastroenterological care. GI investigation including esophagogastroduodenoscopy, colonoscopy, capsule endoscopy, and CT enterography is usually negative for IBD. Nonspecific colitis may be found occasionally. Another distinguishable feature in YAOS is spongiotic dermatitis, which is extremely rare in CD.^[33]^ By contrast, cutaneous presentations in CD patients are pyoderma gangrenosum and erythema nodosa that are absent in YAOS. Up to 30% of CD patients carry NOD2 variants, commonly NOD2 1007fs, G908R, and R702W.^[31,34]^ There are differences in other aspects between CD and YAOS.

## Treatment of Enteropathic Arthritis

### Therapy for IBD in General

Treatment of IBD corresponds to the breadth of its presentations, including various pharmacotherapies ranging from steroids to biologics targeting pathways implicated in disease. Biologics approved for CD and UC in the United States include TNF-alpha inhibitors (adalimumab and infliximab), anti-integrins (vedolizumab), and IL12/IL23 inhibitors (ustekinumab).^[35]^ According to the ACCENT I trial, infliximab for the treatment of CD has been shown to have an 81% clinical response rate by week 4^[36]^ and a 39% clinical remission rate by week 30.^[37]^ For the treatment of UC with infliximab, the clinical remission rate was 33%–39% by week 8 and 20% by week 54 according to the ACT I and II trials.^[38]^ For CD, adalimumab has been shown to have a clinical remission rate of 36% at week 4 by the CLASSIC I trial^[39]^ and a clinical remission rate of 40% at week 26 by the CHARM trial.^[40]^ For UC patients treated with adalimumab, the clinical remission rate was 17% by week 8 and 17% by week 52 based on the ULTRA-2 trial.^[41]^For CD, vedolizumab has shown a clinical remission rate of 15% by week 6 and 39% by week 52 according to the GEMINI II trial.^[42]^ For UC patients treated with vedolizumab, the clinical remission rate was 17% at week 6 and 42% at week 52 per the GEMINI I trial.^[43]^ For CD, ustekinumab has shown a clinical remission rate of 19% for anti-TNF-α nonresponders or 35% for those who had not failed anti-TNF-α by week 6, and a clinical remission rate of 53% at week 44 for anti-TNF-α nonresponders.^[44]^ For UC, the clinical remission rate at 8 weeks was 16%, and that at 44 weeks was 38%–44% per the UNIFI trial.^[42]^

Other therapies span antibiotics, endoscopic procedures, and surgeries. Antibiotics have been shown to have benefit in CD owing to an unclear mechanism potentially by altering gut flora.^[45]^ Endoscopic therapies including endoscopic balloon dilation, electro-incision, or stents have emerged as important ways of managing CD strictures.^[46]^ Patients with medically or endoscopically refractory IBD usually require surgical intervention. Commonly performed surgeries for CD are bowel resection and anastomosis, stricturoplasty, incision and drainage of abscess, and fecal diversion.^[47]^ The standard surgical treatment modality for UC is total proctocolectomy and ileal pouch–anal anastomosis.^[47]^ EIMs including arthropathy may persist after the surgical procedures for IBD.^[48]^

### Therapy for Enteropathic Arthritis

Because of the similar joint involvement between enteropathic arthritis and other SpA (axial SpA and PsA), treatment modalities for IBD-associated arthropathy are extrapolated from studies in other SpA.^[15,16]^ Nonpharmacologic therapies, such as patient education, exercise, physical therapy, rehabilitation, patient associations, and self-help groups, are helpful.^[16]^

Nonsteroidal anti-inflammatory drugs (NSAIDS) have a therapeutic role in treatment of SpA.^[49]^ However, potential NSAID-induced IBD exacerbation has been a concern for healthcare providers.^[50]^ A comprehensive review of various articles was carried. Kefalakes *et al*.^[51]^ showed conflicting but substantial evidence for exacerbation of IBD symptoms after NSAID use. By contrast, a meta-analysis study^[52]^ and a randomized clinical trial^[53]^ showed no correlation between NSAID use and IBD relapses. The American College of Rheumatology (ACR) strongly recommends the treatment of enteropathic arthritis with NSAIDs over no treatment with NSAIDs, and there is no preference on choice of a particular NSAID.^[54]^

Glucocorticoids (GCs) can be used in management of both IBD and arthritis.^[16]^ Intra-articular and systemic GCs have therapeutic roles in enteropathic oligoarticular peripheral arthritis.^[16]^ For systemic use, low doses of steroids should be given for a short period of time.^[55]^ Systemic steroid use should be avoided for axial involvements because of a lack of benefit and risk for adverse effects.^[54]^ Studies have shown the therapeutic role of budesonide for inflammatory arthritis.^[56,57]^

## Synthetic Disease-Modifying Antirheumatic Drugs (Sdmards)

This group of medications includes methotrexate, sulfasalazine, and azathioprine. They are effective in the treatment of peripheral inflammatory arthritis and are less useful for axial manifestation in enterohepatic arthritis.^[58]^ Methotrexate is a dihydrofolate reductase inhibitor and has anti-inflammatory and antiproliferative effects. It showed efficacy in an observational study in the treatment of peripheral arthritis associated with UC.^[59]^ However, it is not effective in management of axial arthritis.^[15]^ Sulfasalazine is a 5-ASA prodrug and showed efficacy in a study of 600 patients with SpA. It was effective, safe, and well tolerated in controlling axial and peripheral articular symptoms in 59% of patients in the treatment group versus 43% in the placebo group.^[60]^ However, according to 2019 ACR guidelines for management of active ankylosing spondylitis, sulfasalazine should be considered only in patients with prominent peripheral arthritis.^[61]^ Sulfasalazine also has a role in the management of UC but is less effective in CD.^[62]^ Azathioprine is a 6-MP prodrug, decreases de novo synthesis of purines, and has anti-inflammatory and antiproliferative effects. It also has a role in the management of peripheral arthritis in rheumatic disease, but there is no sufficient evidence for the management of axial SpA.^[16]^

## Biologic Disease-Modifying Antirheumatic Drugs (Bdmards)

### TNF-Alpha Inhibitors

TNF-α inhibitors are proven to have therapeutic benefit for SpA management according to the pivotal phase III trials. These TNF-α inhibitors are infliximab, etanercept, adalimumab, certolizumab, and golimumab.^[16]^

Adalimumab is a fully humanized monoclonal antibody and displayed clinical improvement in the VITALITY study of health and disability of 164 biologic-naive patients with diagnosis of RA, CD, and PsA over 6 months of adalimumab use.^[33]^ In a clinical trial of 42 CD patients with at least one EIM, adalimumab was effective in reducing EIM associated with CD over 6 months of trial.^[34]^ Infliximab is a chimeric mouse–human monoclonal antibody. In a study of 24 patients with SpA and CD in terms of the efficacy and tolerability of infliximab, there was clinical improvement in both GI and articular symptoms.^[63]^ Golimumab is a fully human IgG1κ monoclonal antibody. In an observational study of 12 patients with active CD and SpA who failed to respond or were intolerant of inhibitors, GI and rheumatologic disease activities were evaluated over 2 years. In a total of 9 patients treated with golimumab, there was clinical improvement in CD activity, and SpA activity assessment revealed a significant reduction in the tender joint count after 6 months, 12 months, and 24 months of treatment. The swollen joint count, pain, SpA disease activity, and disability all decreased in several patients with good safety. This study suggest that golimumab may be an alternative therapeutic option for enteropathic spondyloarthropathy in CD patients who are not responsive to other TNF inhibitors.^[64]^ Certolizumab is a Fab fragment of a recombinant, humanized anti-TNF monoclonal antibody. Studies have shown effectiveness in the treatment of AS, PsA, and CD.^[16,58]^ Etanercept is a bioengineered dimeric soluble TNF receptor blocker. It is not effective for the treatment of CD and is also associated with higher disease activity.^[65,66,67]^ In an observational study, it was effective in alleviating articular symptoms in SpA but showed no improvement in GI symptoms.^[68]^

## Other Biologics

Ustekinumab is monoclonal antibody against IL-12/23. It is effective for both PsA and CD.^[69,70]^ Extrapolating from the data on PsA, this drug is an option for patients with IBD-associated arthritis. However, it is not effective for patients with axial involvement.^[16]^ Secukinumab is a human monoclonal antibody that binds to IL-17a and is ineffective for the management of IBD. Instead, this drug could induce new onset of IBD and flare-ups.^[71]^ Tocilizumab is a humanized monoclonal antibody against the IL-6 receptor and is ineffective for SpA and IBD. It has been associated with intestinal ulcers and perforation, and therefore is not an ideal choice for patients with IBD.^[72]^ Vedolizumab is a monoclonal antibody against integrin α4β7 and specifically targets the GI tract, demonstrating efficacy for the treatment of both UC and CD compared to the placebo. The drug has a minor efficacy in the management of joint symptoms.^[73]^ Vedolizumab is not routinely used in the management of patients with IBD-associated arthritis.^[16]^

## Agents of Small Molecule

Janus kinase (JAK) inhibitors are oral, small molecules, inhibiting different JAK kinase pathways. Several molecules have been developed, including tofacitinib, upadacitinib, baricitinib, and filgotinib, and many others are being developed. Tofacitinib mainly inhibits JAK 1/JAK 3 and is a weak JAK 2 inhibitor. It is efficacious for inducing remission of moderately to severely active UC. It is approved by the FDA to treat UC.^[74]^ It can be used in UC and axial SpA patients. A recent phase III randomized clinical trial of 269 patients with ankylosing spondylitis comparing tofacitinib 5 mg twice daily with placebo showed improvement as early as 2–4 weeks after initiating therapy. An ASAS40 response at week 16 was more likely with tofacitinib (40.6% versus 12.5%).^[75]^ Currently, tofacitinib is the first Jak inhibitor which is approved by the FDA for axial SpA management. Upadacitinib is a selective JAK 1 inhibitor. In a phase II RTC trial of patients with CD, upadacitinib induced endoscopic remission in a significant proportion of patients compared with the placebo.^[76]^ In a phase IIb trial of UC patients, treatment with upadacitinib over 8 weeks was more effective than the placebo for inducing remission in patients with moderately to severely active UC.^[77]^ Filgotinib is an oral selective JAK1 inhibitor. In a RTC double-blinded, placebo control trial (TORTUGA), it showed efficacy and safety for the treatment of patients with active axial SpA who failed to respond to first-line pharmacological therapy with NSAIDs.^[78]^ In another study (SELECTION study) of UC patients, a phase IIb/III RCT for induction and maintenance therapy, filgotinib showed efficacy in inducing and maintaining clinical remission compared with the placebo in patients with moderately to severely active UC.^[79]^

In summary, IBD is a chronic inflammatory disease affecting the GI tract and extraintestinal organs. Due to its various presentations, IBD can share similar clinical phenotypes and genotypes with other autoimmune and autoinflammatory diseases. Differential diagnoses of IBD should, therefore, be extended. Among the EIMs of IBD, enteropathic arthritis is common, involving both peripheral and axial joints. Therapy for IBD, enteropathic arthritis in particular, is broad, including conventional and biologic disease-modifying antirheumatic drugs (DMARDs), as well as small molecules. There are some other new drugs under investigation and development, which may bring more therapeutic options in the pipeline.
